# Extracellular ATP/adenosine dynamics in the brain and its role in health and disease

**DOI:** 10.3389/fcell.2023.1343653

**Published:** 2024-01-18

**Authors:** Eiji Shigetomi, Kent Sakai, Schuichi Koizumi

**Affiliations:** ^1^ Department of Neuropharmacology, Interdisciplinary Graduate School of Medicine, University of Yamanashi, Chuo, Japan; ^2^ Yamanashi GLIA Center, Interdisciplinary Graduate School of Medicine, University of Yamanashi, Chuo, Japan

**Keywords:** ATP, adenosine, purinergic receptor, neurological disease, astrocytes, microglia, genetically encoded sensors, seizure

## Abstract

Extracellular ATP and adenosine are neuromodulators that regulate numerous neuronal functions in the brain. Neuronal activity and brain insults such as ischemic and traumatic injury upregulate these neuromodulators, which exert their effects by activating purinergic receptors. In addition, extracellular ATP/adenosine signaling plays a pivotal role in the pathogenesis of neurological diseases. Virtually every cell type in the brain contributes to the elevation of ATP/adenosine, and various mechanisms underlying this increase have been proposed. Extracellular adenosine is thought to be mainly produced via the degradation of extracellular ATP. However, adenosine is also released from neurons and glia in the brain. Therefore, the regulation of extracellular ATP/adenosine in physiological and pathophysiological conditions is likely far more complex than previously thought. To elucidate the complex mechanisms that regulate extracellular ATP/adenosine levels, accurate methods of assessing their spatiotemporal dynamics are needed. Several novel techniques for acquiring spatiotemporal information on extracellular ATP/adenosine, including fluorescent sensors, have been developed and have started to reveal the mechanisms underlying the release, uptake and degradation of ATP/adenosine. Here, we review methods for analyzing extracellular ATP/adenosine dynamics as well as the current state of knowledge on the spatiotemporal dynamics of ATP/adenosine in the brain. We focus on the mechanisms used by neurons and glia to cooperatively produce the activity-dependent increase in ATP/adenosine and its physiological and pathophysiological significance in the brain.

## Introduction

Intracellular ATP is the main cellular currency of energy in the brain and is used for various functions including neurotransmitter release, maintenance of ionic gradients, and intracellular transport (Erecińska and Silver, 1989). Extracellular ATP and its metabolite adenosine are key neuromodulators in the central nervous system (Burnstock, 2007). ATP and adenosine activate specific receptors. ATP activates P2 receptors, which are divided into the following two subclasses: P2X (P2X1-7) and P2Y (P2Y_1_,_2_,_4_,_6_,_11_,_12_,_13_,_14_). P2X receptors are ligand-gated channels (Khakh and North, 2006), whereas P2Y receptors are G-protein-coupled receptors (GPCRs). Adenosine activates P1 (A_1_, A_2A_, A_2B_, A_3_) receptors, which are GPCRs (Fredholm et al., 2005). Extracellular ATP and adenosine concentrations are regulated by multiple channels, enzymes and transporters. ATP is rapidly metabolized in the extracellular space by ectonucleotidases including E-NTPDases, E-NPPases, and alkaline phosphatases (Zimmermann, 2000; Zimmermann et al., 2012). This limits the availability of ATP for activating P2 receptors. Furthermore, ATP is degraded to adenosine via ADP or AMP by ecto-5′-nucleotidase (Zimmermann, 2000; Zimmermann et al., 2012). ATP is one of the major sources of extracellular adenosine. Therefore, the increase in ATP augments not only P2 receptor signaling, but P1 signaling as well (Kato et al., 2004). The molecules that regulate purinergic signaling, including receptors, channels, enzymes and transporters, are expressed by both neurons and glia. Some of these molecules are expressed in specific cell types (e.g., P2Y_12_ receptor in microglia). Accumulating evidence indicates that purinergic signaling plays a key role in neuron–glial communication in the brain (Fields and Burnstock, 2006).

ATP and adenosine are physiologically important neurotransmitters/neuromodulators to activate P2 and P1 receptors, respectively, in the brain (Latini and Pedata, 2001; North and Verkhratsky, 2006; Pankratov et al., 2009; Khakh and North, 2012). For example, ATP is a chemical mediator of chemosensation in the brainstem (Gourine et al., 2005), and adenosine regulates the sleep–wake cycle (Basheer et al., 2004) as well as learning and memory (Rebola et al., 2008). In pathophysiological conditions such as seizure, ischemia and traumatic brain injury, both ATP and adenosine are elevated and play pivotal roles in the diseases (Beamer et al., 2021; Schädlich et al., 2023). ATP is released from damaged tissue and during inflammation to alert surrounding cells in the brain (Rodrigues et al., 2015). Abnormalities in ATP and adenosine regulation are found in neurological (Pietrowski et al., 2021) and psychiatric (Illes et al., 2019) disorders. Adenosine is generally thought to play a protective role in neurons via hyperpolarization and the suppression of synaptic transmission (Dunwiddie and Masino, 2001; Agostinho et al., 2020). However, ATP and adenosine can play both protective and detrimental roles.

Adenosine is originally thought to be released from neurons in an activity-dependent manner. However, recent findings suggest that adenosine is also derived from ATP released from astrocytes and that microglia play a major role in the conversion of ATP to adenosine. ATP is released from almost every cell type via distinct pathways (Taruno, 2018; Dale et al., 2023). Therefore, purinergic signaling is far more complex than previously thought.

In this review, we focus on the mechanisms of purinergic signaling. To unravel the complexity, an in-depth understanding of the spatiotemporal dynamics of ATP/adenosine is essential (Dale, 2021a). We describe current methods of evaluating the spatiotemporal dynamics of ATP/adenosine (Wu and Li, 2020; Dale, 2021b), with a focus on recent studies that have advanced our knowledge of purinergic signaling in the brain.

## Methods of measuring extracellular ATP/adenosine

Several methods have been developed for measuring extracellular purines. Fluorescence-based sensors and sniffer-cell-based methods offer better spatial information than electrode sensors or bioluminescence-based methods. Direct measurement of ATP/adenosine provides useful information for uncovering the complexity of purinergic signaling, although none of the available methods allow for the monitoring of extracellular ATP/adenosine spatiotemporal dynamics with high sensitivity. We briefly summarize the methods below. Comprehensive reviews by other groups are available (Wu and Li, 2020; Dale, 2021b).

### Bioluminescence

Firefly luciferase, an ATP-consuming enzyme that emits light in the presence of luciferin and oxygen, is widely used to measure extracellular ATP. This method is highly specific for ATP; however, the number of emitted photons is low, which requires a longer collection time than other methods. In many studies, the assay is used to measure ATP accumulated in culture media, in artificial cerebrospinal fluid (aCSF), or in extracellular fluid collected from the brain by microdialysis. Therefore, temporal resolution of bioluminescence methods is very low. Using a photon counting camera, ATP release can be measured using luciferase-based bioluminescence. This method can detect the spreading of ATP released by cultured glial cells (Wang et al., 2000; Newman, 2001; Koizumi et al., 2003). The temporal resolution is limited by the turnover of luciferase (Dale, 2021b). pmeLUC, a plasma membrane-targeted luciferase, allows for the monitoring of ATP *in vivo*, and this method can be used to assess ATP distribution at the whole-animal level (Pellegatti et al., 2008; Wilmes et al., 2022).

### HPLC

Adenosine levels in culture media and extracellular fluid have been detected and measured by HPLC (Porkka-Heiskanen et al., 2000). Although this method has high specificity for adenosine, the samples are collected from microdialysis, therefore temporal resolution is low.

### Enzyme-based biosensors

Nicholas Dale and their colleagues have developed enzyme-based biosensors (Dale et al., 2000; Gourine et al., 2005; Wall and Dale, 2013; Dale, 2021b) that produce H_2_O_2_, which is detected electrochemically by a platinum electrode. In one of the enzyme-based ATP biosensor, two enzymes are coated on the electrode surface—glycerol kinase and glycerol-3-phosphate oxidase. Glycerol is required for this sensor to work, but the reaction only requires ATP, and therefore, the sensor is insensitive to ADP and adenosine. For adenosine biosensors, three enzymes are coated on the electrode surface—adenosine deaminase, purine nucleoside phosphorylase, and xanthine oxidase. To detect ATP or adenosine-specific responses, an enzyme-null sensor (control sensor) is placed near the biosensor. The sensitivity of detection is in the nanomolar level, which is relatively high compared with other methods. The biosensors offer relatively fast measurement (in the order of seconds) of extracellular ATP and adenosine. The diameter of the biosensors is 7–50 μm. Kazuaki Sawada and their colleagues developed a complementary metal oxide semiconductor (CMOS) imaging sensor to measure H^+^. By coating apyrase, an ATP-degrading enzyme, on the CMOS imaging sensor, it can provide spatiotemporal information on ATP release in brain slices with high temporal resolution (Doi et al., 2021). These enzyme-based methods offer high temporal resolution of ATP/adenosine dynamics. Although these sensors are placed near the tissue but not in the tissue, they may not always be close to the point of release of ATP/adenosine, and it is likely that they report accumulation of purines released from multiple sites.

### Fast-scan cyclic voltammetry (FSCV)

Jil Venton and their colleagues developed FSCV, employing a carbon microelectrode (diameter, 7 μm) to detect adenosine directly on a sub-second time scale (Swamy and Venton, 2007; Nguyen and Venton, 2015). FSCV is an electrochemical method for measuring changes in electroactive molecules, such as adenosine, which undergoes three sequential two-electron oxidations by FSCV. The temporal resolution of this method is in the order of hundreds of milliseconds, which allows for the detection of rapid changes in adenosine. However, similar to enzyme-based biosensors, the placement of multiple sensors is required to acquire spatial information. This method can detect spontaneous adenosine release in addition to rapid adenosine changes induced by activity *in situ* (Nguyen et al., 2014). FSCV offers high specificity for adenosine, but not adenosine metabolites. FSCVs employing carbon microelectrodes can be inserted into the tissue *in situ* and *in vivo*, thereby permitting the measurement of adenosine at the site of interest.

### Sniffer-based methods

To detect ATP electrophysiologically, an ATP-gated ion channel, P2X2 or P2X7, is expressed in cultured cells, such as HEK293T, and ATP-mediated currents are recorded using the patch-clamp method (Hayashi et al., 2004; Lalo et al., 2014). Recordings can be made on a whole cell or a small patch of the plasma membrane. The temporal resolution of this method is in the order of milliseconds. If the sniffer cell/patch is close enough to the release site, exocytotic events can be recorded (Lalo et al., 2014). P2X receptors are Ca^2+^-permeable channels, and some of the P2Y channels are Gq-GPCRs, whose activation increases intracellular Ca^2+^. Therefore, the combination of sniffer cells with Ca^2+^ imaging can be used to monitor ATP (Haas et al., 2006). To monitor adenosine by Ca^2+^ imaging, A_1_ receptor and Gqi, a chimeric Gq alpha subunit that couples with the A_1_ receptor, are co-expressed in HEK293 cells (Yamashiro et al., 2017). To measure the spatiotemporal dynamics of purines, brain tissues are placed on the sniffer cell cultures. These methods are highly sensitive for ATP/adenosine because the functional receptor is used to detect the purines. Similar to biosensors, it is likely that this method reports accumulation of extracellular purines released from multiple sites.

### Fluorescence sensors

Several groups have developed fluorescence-based optical sensors. Fluorescence sensors are bright and technically easier to use than the luciferase-based method, which requires a constant supply of luciferin. Genetically-encoded neurotransmitter or neuromodulator sensors (GENI) can be expressed under the control of cell-type specific promoters, thereby allowing cell-type-specific monitoring of extracellular chemicals (Wu et al., 2022a). By labeling specific sites/cells in the tissues, these sensors can monitor spatial dynamics of ATP/adenosine at the subcellular level.

Baljit Khakh and their colleagues created P2X2-cam to monitor extracellular ATP (Richler et al., 2008a). P2X2-cam is a P2X2 subunit tagged with yellow cameleon (cam), a fluorescence resonance energy transfer (FRET)-based Ca^2+^ sensor protein. When P2X2 is activated by ATP, Ca^2+^ enters the cytosol through the P2X2 receptor pore to bind cam thereby increasing FRET efficiency.

ecATeam3.10 is a genetically-encoded FRET-based sensor for detecting extracellular ATP (Conley et al., 2017). ecATeam3.10 is derived from ATeam, which was originally developed by Hiroyuki Noji, Hiromi Imamura and their colleagues as a sensor to visualize ATP levels within living cells and subcellular components (Imamura et al., 2009). The epsilon subunit of the bacterial FoF_1_ ATPase is used as an ATP-sensing domain. CFP and YFP bind to both sides of the FoF_1_ ATPase. Baljit Khakh, Loren Looger and their colleagues designed iATPSnFR1.0, a genetically-encoded single-wavelength ATP sensor, which contains the epsilon subunit of the *Bacillus* PS3 FoF_1_ ATPase and circularly-permutated GFP (cpGFP) (Lobas et al., 2019). These FoF_1_ ATPase-based sensors are selective for ATP; however, their sensitivity is relatively low.

Kenzo Hirose and their colleagues developed ATPOS, which is the Cy3-conjugated epsilon subunit of *Bacillus* PS3 FoF_1_, for ATP detection (Kitajima et al., 2020). ATPOS is bright and has the highest sensitivity among sensors using the epsilon subunit of FoF_1_ ATPase. To visualize ATP dynamics in the tissue, ATPOS is tagged with a nontoxic subunit of botulinum neurotoxin. Because of this, ATPOS is unable to target specific sites/cells. These sensors, based on the epsilon subunit of FoF_1_, weakly detect ADP, but do not detect ADO when ATPOS is expressed on the cell surface (Lobas et al., 2019).

Yulong Li, Zhaofa Wu and their colleagues developed genetically-encoded G protein-coupled receptor-activation-based (GRAB) sensors for ATP (GRAB_ATP_) and adenosine (GRAB_ADO_) detection (Peng et al., 2020; Wu et al., 2022b; Wu et al., 2023). Both sensors are GFP-based single-wavelength sensors. GRAB_ATP_ is based on the human P2Y_1_ receptor, whereas GRAB_ADO_ is based on the human A_2A_ receptor (Peng et al., 2020). Both of these sensors’ responses can be blocked by specific antagonists. MRS2500 blocks GRAB_ATP_, while ZM-241385 blocks GRAB_ADO_. The ability to block the sensors is useful for monitoring basal purine concentration (Wu et al., 2023). In fact, the basal fluorescence of GRAB_ADO1.0_, a high-affinity adenosine detector, is reduced by ZM-241385 (Wu et al., 2023). Because GRAB_ATP_ is based on the P2Y_1_ receptor, it also responds to ATP and ADP, without distinguishing between the two (Wu et al., 2022b). GRAB_ADO1.0_ permits analysis of adenosine dynamics in the sleep–wake cycle by *in vivo* fiber photometry (Peng et al., 2020). GRAB_ATP1.0_ expressed in astrocytes detects the spontaneous release of ATP, some of which seems to occur locally (Hatashita et al., 2023). ATP1.0-L sensor, an ATP sensor with lower affinity and faster kinetics, may be useful to detect local release of ATP (Wu et al., 2022b).

## Extracellular ATP/adenosine increases induced by neuronal activity

Nearly every cell type is equipped with mechanisms for the production and release of ATP/adenosine. To understand cell-to-cell communication via ATP/adenosine, it is critical to know when, where and how ATP/adenosine is produced and released extracellularly. Here, we focus on the activity-dependent release of ATP/adenosine and the underlying regulatory mechanisms, because activity-dependent of ATP/adenosine release is well-known and well-studied in many brain regions. Also, this type of ATP/adenosine release is likely to be physiologically relevant. In most reported studies, activity-dependent release/increase of ATP/adenosine is induced by electrical stimulation of nerve fibers or direct depolarization of neurons by electrophysiological or optogenetic methods. The mechanisms of ATP/adenosine release may differ according to brain region (Porkka-Heiskanen et al., 2000; Latini and Pedata, 2001; Lee and Venton, 2018), but for simplicity, we will focus on the hippocampus.

### ATP release from neurons

Electrical burst stimulation (300 Hz for 50 ms, 2-s intervals for 30 s), but not low-frequency stimulation or glutamate exposure, of the Schaffer collateral causes ATP increase into the aCSF, measured using bioluminescence-based methods (Wieraszko et al., 1989). Although the ATP increase was not observed in Ca^2+^-free aCSF, the source of the ATP was not demonstrated in their study. However, another study showed that electrical stimulation may cause artifactual ATP release via pores created by the stimulation (i.e., electroporation) (Hamann and Attwell, 1996). Therefore, it is important to verify that the ATP increase is not an artifact of the method employed.

**FIGURE 1:**
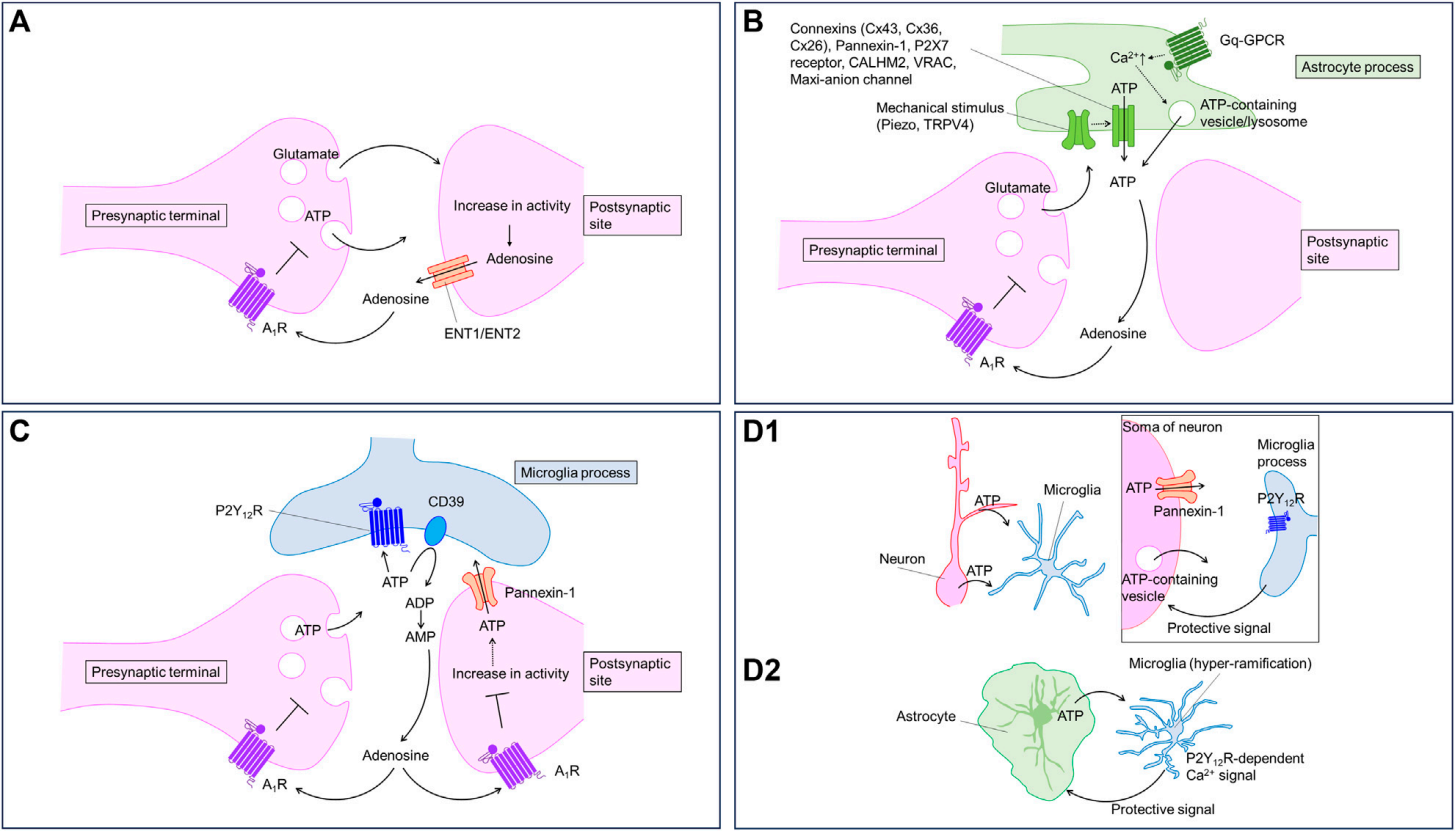
Purinergic signaling regulated by neurons and glia. Neurons and glia communicate with each other using purinergic signaling. Virtually every type of cell can release, receive, and contribute to purinergic signaling; therefore, the timing and location of these events are important. The cartoons show proposed models of how purinergic signaling modulates neuron-glia communication in the brain. **(A)** Neurons release ATP from presynaptic terminals and adenosine from postsynaptic sites in an activity-dependent manner. Adenosine activate A_1_ receptor to inhibit synaptic transmission and to hyperpolarize neurons. **(B)** Astrocytes release ATP in response to several stimuli including neuronal activities. Astrocytic ATP is converted to adenosine activating presynaptic A_1_ receptor to inhibit synaptic transmission. Astrocytes can release ATP through multiple pathways. **(C)** Neuronal ATP activate P2Y_12_ receptors in microglial processes. The ATP is converted to adenosine to decrease neuronal excitability via A_1_ receptor. **(D)** Neuronal **(D1)** or astrocytic **(D2)** ATP activates P2Y_12_ receptors in microglial processes. Microglia, in turn, send protective signals to neurons **(D1)** or astrocytes **(D2)**.

Activity-dependent release of ATP is also demonstrated indirectly by electrophysiological recording of postsynaptic currents mediated by P2X receptors in the hippocampus (Pankratov et al., 1998; Mori et al., 2001), the cortex (Pankratov et al., 2002), locus coeruleus (Nieber et al., 1997) and the spinal cord (Bardoni et al., 1997). However, the P2X receptor-mediated synaptic current is small compared with the AMPA/KA receptor-mediated currents. Thus, the contribution of P2X receptor to the neuronal excitability is relatively small compared to that of AMPA/KA receptor. Electrophysiological recordings in the absence of neuronal activity have demonstrated the quantal release of ATP in several brain regions (Edwards et al., 1992; Pankratov et al., 2006). The data suggest that ATP is released from presynaptic terminals (Figure 1A). Sawada et al. (2008) showed that vesicular nucleotide transporter (VNUT) participates in ATP uptake into vesicles. VNUT immunoreactivity is detected in axons, dendritic spines and astrocytes (Sawada et al., 2008; Larsson et al., 2011). Although VNUT-dependent ATP release has not been shown in hippocampal tissues, neuronal VNUT has been reported to contribute to ATP elevation in the spinal cord after spinal nerve injury (Masuda et al., 2016). In hippocampal neuronal cultures, electrical stimulation causes ATP responses detected by P2X2-cam. The ATP responses are correlated with the number of stimuli. Pharmacological experiments suggest that neurons release ATP via exocytosis (Richler et al., 2008b). The activity-dependent release of ATP from neurons can be indirectly detected by measuring Ca^2+^ signals in astrocytes (Bowser and Khakh, 2004; Jourdain et al., 2007; Tang et al., 2015; Shigetomi et al., 2018). However, there has been no attempt to directly measure the activity-dependent release of ATP from neurons *in situ* or *in vivo*, although indirect measurement of the activity-dependent release of ATP from neurons has been reported.

Neurons can also release ATP from axons via volume-regulated anion channels (VRACs) (Fields and Ni, 2010). Recently, LRRC8A-containing VRAC is shown to mediate ATP release in *Xenopus* oocytes (Gaitán-Peñas et al., 2016). Furthermore, it has been suggested that neurons release ATP from cell somata or dendrites via pannexin-1, a similar channel (Dissing-Olesen et al., 2014; Eyo et al., 2014), or VNUT (Cserép et al., 2020) (Figure 1D1). However, whether these pathways contribute to activity-dependent ATP release from neurons remains unknown. Pannexin-1 may be relevant to ATP release in pathological conditions (described below). In addition, pannexin-1 is expressed in oligodendrocytes (Domercq et al., 2010) and epiplexus cells (Maslieieva and Thompson, 2014) contributing to ATP release from these cell types.

### ATP release from astrocytes

ATP is a major gliotransmitter in astrocytes. Early studies demonstrated that ATP can be released by poking astrocytes using a glass pipette in culture (Guthrie et al., 1999; Coco et al., 2003; Koizumi et al., 2003; Newman, 2003; Bowser and Khakh, 2007). ATP release was measured by luciferase-based assay and Ca^2+^ imaging. The vesicular release pathway may contribute to mechanical pressure-induced ATP release from astrocytes in culture (Coco et al., 2003; Bowser and Khakh, 2007); however, how mechanical pressure triggers the vesicular release is unknown. Based on Ca^2+^ imaging and pharmacological studies, Turovsky et al. (2020) show that mechanical pressure, assessed using magnetic particles, causes ATP release from cultured astrocytes via an interaction between TRPV4 and Cx43 channels. TRPV4 seems important for mechanosensation and Cx43 connexin hemichannel functions in ATP release pathways (Cotrina et al., 1998; Stout et al., 2002). Recently, Chi et al. (2022) show that Piezo1, a specialized mechanosensor, mediates mechanical pressure-induced ATP release and could be upstream of TRPV4-Cx43 (Figure 1B). Using astrocytic Piezo1 conditional knockout (cKO) mice and GsMTx4, a Piezo1 channel blocker, the authors showed that Pizeo1 channel activity is required for theta burst (TBS) stimulation-induced long-term potentiation (LTP). ATP hydrolysis by apyrase impaired the LTP, while the addition of ATP rescued LTP in the Pizeo1 cKO, suggesting that ATP release by mechanotransduction through astrocytic Piezo1 is critical for LTP (Pankratov et al., 2009; Chi et al., 2022). It is unclear how TBS increases Piezo1 activity or impacts ATP release. Mechanotransduction-induced ATP release is likely to be mediated via TRPV4 and connexins. It would be interesting to visualize ATP dynamics during TBS. In addition to mechanical pressure-induced ATP release, connexin hemichannels have been shown to regulate ATP release induced by high-frequency stimulation, which reduces extracellular Ca^2+^ levels. The connexin hemichannels open in response to a decrease in extracellular Ca^2+^, permitting ATP release (Torres et al., 2012).

Astrocytes release ATP in a Ca^2+^-dependent manner (Figure 1B). Because astrocytes display Ca^2+^ responses upon neurotransmitter stimulation (Bazargani and Attwell, 2016; Shigetomi et al., 2016), a Ca^2+^-dependent mechanism likely mediates activity-dependent ATP release. Consistent with this, Cao et al. showed lower ATP levels in the media of astrocyte cultures prepared from mice deficient in IP_3_ receptor type 2, which plays a major role in Ca^2+^ release from the endoplasmic reticulum (Cao et al., 2013). Although Ca^2+^ likely regulates many pathways of ATP release, exocytosis is the most attractive. Pascual et al. (2005) showed that blocking vesicular release using dominant negative SNARE domain (dnSNARE) specifically in astrocytes decreases adenosine-mediated synaptic depression. Pharmacological inhibition of ecto-nucleotidase, which converts ATP to AMP, induces P2 receptor-mediated (i.e., ATP-mediated) synaptic regulation, suggesting that astrocytes release ATP rather than adenosine via exocytosis. Lysosomes containing VNUT may contribute to the exocytotic ATP release (Zhang et al., 2007; Oya et al., 2013). Although VNUT is expressed in astrocytes, and manipulation of VNUT expression causes biochemical and behavioral changes in mice (Kinoshita et al., 2018), recent omics analyses have revealed a lack of molecular machinery for vesicular release in astrocytes (Chai et al., 2017; Soto et al., 2023). Specialized astrocytes could be equipped with the release machinery for ATP, similar to that reported for glutamate (de Ceglia et al., 2023). *In vivo* studies on vesicular release and its machinery are needed for a better understanding of the role of vesicular ATP release. Direct measurement of astrocytic ATP release might be useful in these studies.

Astrocytes can also release ATP via other pathways, including pannexins (Suadicani et al., 2012), P2X7 receptor (Sylvia et al., 2006), VRAC (Fujii et al., 2017), CALHM2 (Ma et al., 2018), and maxi-anion channels (Liu et al., 2008). There are excellent reviews on channel-mediated ATP release mechanisms (Dale et al., 2023).

All the evidence on astrocytic ATP release discussed above is based on ATP measurements using luciferase-based methods and indirect measurements using either Ca^2+^ imaging in astrocytes or electrophysiological recordings combined with pharmacological intervention. Therefore, it is unclear where and how much ATP is released in an activity-dependent manner. However, direct measurement of ATP release using fluorescence-based sensors has recently begun to uncover the complexity of ATP dynamics *in situ* and *in vivo*. Kitajima et al. showed high K^+^-evoked ATP wave-like propagation, at a rate of 2 mm/min, in the cortex of mice (Kitajima et al., 2020). The mechanism underlying the ATP waves was not identified, but similar experiments using hippocampal slices demonstrate a role of P2X7 receptors and pannexin (Heinrich et al., 2012). Lu et al. expressed GRAB_ATP1.0_ in astrocytes in the medial prefrontal cortex of mice to monitor ATP dynamics using fiber photometry. After social defeat stress, the ATP increase during forced social interaction was reduced in mice lacking astrocytic glucocorticoid receptor (GR), whose activation modulates astrocytic ATP release. GR-dependent ATP release is mediated by the PI3 kinase–Akt pathway, which regulates lysosomal exocytosis (Lu et al., 2022). GR-dependent ATP release is also found in spinal astrocyte cultures, in which pannexin-1 plays a key role (Koyanagi et al., 2016).

Another *in vivo* imaging study using GRAB_ATP1.0_ revealed that dopamine activates α1-Adrenergic receptors in astrocytes in the prefrontal cortex *in vivo*, causing an increase in ATP events (Pittolo et al., 2022). Dopamine increases Ca^2+^ signal events in astrocytes, suggesting a Ca^2+^-dependent mechanism underlies the ATP events. Hatashita et al. examined spontaneous ATP dynamics *in situ* in the presence of TTX and in anesthetized mice using GRAB_ATP1.0_ (Hatashita et al., 2023). The investigators found spatiotemporally diverse types of spontaneous ATP events. By performing simultaneous Ca^2+^ imaging with GRAB_ATP1.0_ imaging *in situ*, they found that most spontaneous ATP events occurred in a Ca^2+^-independent manner, which is surprising because many previous studies have indicated Ca^2+^-dependent ATP release from astrocytes. Mechanisms underlying evoked and spontaneous ATP release from astrocytes may differ. Moreover, the pharmacological data of Hatashita et al. suggest that multiple mechanisms contribute to the ATP increase near astrocytes, including exocytosis and VRAC. Overall, direct measurements of ATP have started revealing its dynamics *in situ* and *in vivo*; however, the molecular mechanism underlying ATP events is not clear. It is necessary to clarify the underlying mechanisms by knockdown/knockout of signaling molecules that trigger ATP release as well as the components of the ATP release machinery.

### Microglial contribution to ATP increases

Microglia also release ATP, although their contribution to extracellular ATP is less well known than that of neurons or astrocytes. Imura et al. (2013) showed that VNUT contributes to ATP release. VNUT expression is low in cultured microglia; however, the transporter is upregulated by lipopolysaccharide (LPS), indicating its relevance to inflammation. Interestingly, Wu et al. (2022b) showed that intraperitoneal injection of LPS increases localized ATP events (diameter, ∼10 μm) in astrocytes expressing GRAB_ATP_ in the cortex of mice *in vivo*. The ATP events appear within 30 min after the LPS injection and last at least 24 h. The mechanisms underlying the events are unknown. Microglia also have a channel-mediated ATP release pathway. Recently, Chu et al. (2023) showed that SWELL1, a VRAC, contributes to ATP release from microglia, which plays a role in pain-like behavior in the chronic constriction injury model of neuropathic pain.

### Adenosine release from neurons

Adenosine is thought to be directly released from neurons in an activity-dependent manner playing a role in short-term plasticity via A_1_ receptor (Mitchell et al., 1993; Manzoni et al., 1994). Elevating intracellular adenosine induces the release of adenosine, inhibiting excitatory input (Brundege and Dunwiddie, 1996). Ecto-nucleotidase inhibition prevents the exogenous ATP-induced reduction of synaptic transmission, but not activity-dependent short-term depression in the hippocampus, both of which are blocked by adenosine A_1_ receptor antagonism. These findings suggest that direct release of adenosine but not ATP from neurons contribute to the activity-dependent release of adenosine (Brager and Thompson, 2003). Several studies suggest that equilibrate nucleoside transporters (ENTs) mediate activity-dependent adenosine release from neurons. Lovatt et al. showed that action potentials induced by 1-s depolarizing pulses in neurons with whole-cell patch clamp recording cause synaptic depression via A_1_ receptor activation (Lovatt et al., 2012). Adding inosine into the recording pipette to inhibit ENTs blocked depolarization-induced synaptic depression, suggesting that adenosine is released from neurons in an activity-dependent manner. Using adenosine biosensors, Wall and Dale directly monitored activity-dependent adenosine release by electrical stimulation of Schaffer collaterals in the hippocampus (Wall and Dale, 2013). Pharmacological inhibition of ENTs reduced the activity-dependent release of adenosine. Recently, using the GRAB_ADO1.0m_ sensor, Wu et al. showed that optogenetic stimulation of CA3 neurons, whose axon collateral projects CA1 region to form the Schaffer collateral, causes adenosine release from somatodendritic sites of CA1 neurons, and that this effect is blocked by an ENT inhibitor or ENT1 knockdown/ENT2 knockout. In support of this, the investigators found that ENT1 and ENT2 mediated the adenosine release in cultured neurons. L-type voltage gated Ca^2+^ channels are required for the adenosine release (Wu et al., 2023). Na^+^ influx induced by neuronal activity and subsequent Na^+^ efflux through Na^+^/K^+^ ATPase consumes ATP to increase intracellular adenosine, which eventually is released via ENTs (Sims and Dale, 2014). Together, these observations suggest that neurons release adenosine in an activity-dependent manner via ENTs (Figure 1A).

Blockade of postsynaptic ionotropic glutamate receptors also reduces adenosine responses, suggesting that postsynaptic mechanisms are involved in adenosine release (Wall and Dale, 2013; Wu et al., 2023). Consistently, Yamashiro et al. showed that activation of postsynaptic ionotropic glutamate receptors is required for adenosine release induced by electrical stimulation of the Schaffer collateral, monitored using sniffer cells (Yamashiro et al., 2017). However, the role of postsynaptic components is controversial. Using FSCV, Pajski and Venton showed that adenosine release is not blocked by ionotropic glutamate receptor antagonists in hippocampal slices (Pajski and Venton, 2013); however, it should be noted that the pulse number in the FSCV study was much smaller than that used in other studies.

### ATP derived from astrocytes is a source of adenosine

ATP is rapidly converted to adenosine in the extracellular space (Dunwiddie et al., 1997) by ectonucleotidases including CD39 (NTPDase1) and CD39L1 (NTPDase2) (Zimmermann, 2000), and exogenously applied ATP evokes an adenosine receptor-mediated synaptic response, specifically, reduction of excitatory synaptic transmission via A_1_ receptor (Cunha et al., 1996; Kato and Shigetomi, 2001; Masino et al., 2002). ATP is a major gliotransmitter released by astrocytes, and therefore, it is thought that ATP is primarily released from astrocytes, not neurons. However, further study is needed to test this concept, particularly as the spatiotemporal dynamics of ATP/adenosine at the cellular level remain unclear. Recent GRAB sensor imaging using fiber photometry, which lacks subcellular resolution, suggests that adenosine dynamics follow ATP dynamics in the basal forebrain, but that adenosine dynamics is independent of Ca^2+^-dependent ATP release from astrocytes (Peng et al., 2023) but dependent on glutamatergic neuron activities (Peng et al., 2020).

Astrocytic ATP is converted to adenosine contributing to heterosynaptic depression in the hippocampus. Using electrophysiological recording in combination with pharmacological approaches, Zhang et al. (2003) showed that ATP release from astrocytes is induced by non-NMDA receptor-activation. The released ATP is converted to adenosine, thereby contributing to heterosynaptic suppression of excitatory synaptic transmission via presynaptic A_1_ receptors. As discussed above, Pascual et al. (2005) showed that astrocytes release ATP, which is converted to adenosine, thereby suppressing excitatory synaptic transmission and long-term plasticity (Figure 1B).

Using an adenosine biosensor combined with knockout of CD73 (ecto-5′-nucleotidase), an enzyme that converts AMP to adenosine, Wall and Dale showed that the activity-dependent increase in adenosine in hippocampal slices is partially mediated by astrocytic ATP release using dnSNARE mice (Wall and Dale, 2013). Because the blockade of ionotropic glutamate receptors by CNQX/D-AP5 suppressed the increase in adenosine, the authors suggested that NMDA receptor activation in astrocytes may cause ATP release from astrocytes (Wall and Dale, 2013). However, there is no direct evidence that NMDA receptor activation in astrocytes alters extracellular adenosine levels. Recently, GluN2C was reported to be functionally expressed in astrocytes in the hippocampus (Chipman et al., 2021). It would be interesting to investigate whether GluN2C activation induces the release of ATP or adenosine by astrocytes.

Panatier et al. showed that local Ca^2+^ elevation in astrocytes mediated by the mGluR5 receptor facilitates glutamate release from the presynaptic terminals of the Schaffer collateral via the activation of presynaptic A_2A_ receptors. Infusion of the light chain of tetanus toxin into astrocytes prevents the mGluR5-dependent facilitation of glutamate release, indicating that vesicular release machinery is required for the release events. Overall data suggest that local Ca^2+^ elevation in astrocytic processes triggers the local release of ATP, which is degraded by ecto-enzymes into adenosine, which in turn activates presynaptic A_2A_ receptors (Panatier et al., 2011).

Selective manipulation of astrocytes using optogenetic and chemogenetic tools to activate Gq-GPCR signaling pathways also cause adenosine-mediated synaptic inhibition via A_1_ receptors (Corkrum et al., 2020; Iwai et al., 2021). It is suggested that adenosine is derived from ATP released from astrocytes. There are numerous other studies demonstrating that astrocytic ATP is a source of adenosine, which participates in synaptic regulation in several brain regions (see below and review by Kofuji and Araque (Kofuji and Araque, 2021) for more detail). Similar mechanisms also appear to be involved at axons. For example, Lezmy et al. showed that astrocytic ATP is released and converted to adenosine near myelinated axons to regulate their excitability (Lezmy et al., 2021).

### Microglial contribution to adenosine increase

Microglia express several purinoceptors that sense purines derived from environmental changes (Koizumi et al., 2013). Among the purinoceptors, P2Y_12_ receptor plays a central role in detecting extracellular ATP derived from neurons and astrocytes. Microglia sense ATP derived from damaged tissue rapidly and extend their processes to the damaged sites (Davalos et al., 2005; Haynes et al., 2006). An increase in neuronal excitability induced by the activation of neuronal ionotropic glutamate receptors with kainite or NMDA also triggers the release of ATP from dendrites and soma, which in turn elicits microglial process extension towards the release sites via P2Y_12_ receptor (Dissing-Olesen et al., 2014; Eyo et al., 2014). Cserép et al. showed that microglial processes expressing P2Y_12_ receptors preferentially contact the specialized somatic regions of neurons where Kv2.1 and Kv2.2 clusters in the plasma membrane are localized. Furthermore, microglial contact on neuronal soma is dependent on P2Y_12_ receptor and linked with metabolic activity of neuronal mitochondria (Cserép et al., 2020). Loss of P2Y_12_ receptor worsens kainite-induced seizure (Eyo et al., 2014). Pharmacological blockade of P2Y_12_ receptor increases damage by middle cerebral artery occlusion (Cserép et al., 2020). These reports suggest that microglial–neuronal contacts formed in response to neuronal activity or brain insults are neuroprotective (Figure 1D1). However, the effectors that mediate the neuroprotection are unclear. Badimon et al. proposed that adenosine might be a key mediator for the neuroprotection (Badimon et al., 2020). The investigators focused on the striatum, and found that CD39, an enzyme converting ATP to AMP, is mainly expressed in microglia, while CD73, an enzyme converting AMP to adenosine, is mainly expressed in neurons. Microglial depletion decreases adenosine levels and causes hyperexcitability of neurons. Knockout of CD39 specifically in microglia also results in severe drug-induced seizure in mice, which can be mimicked by A_1_ receptor antagonism or P2Y_12_ receptor knockout. These findings suggest that microglia sense ATP released from neurons during the seizure and produce adenosine to suppress neuronal hyperactivity via A_1_ receptors (Figure 1C). CD39 in microglia plays an important role in not only the suppression of neuronal hyperexcitability, but also in homeostasis in microglial processes (Matyash et al., 2017). In addition to converting ATP to adenosine, microglia may also increase extracellular adenosine directly. CX3CL1 increases adenosine in a microglial cell line (Lauro et al., 2008), although the underlying mechanism is not clear.

## Role of extracellular ATP/adenosine in seizure models

As discussed above, most cells in the brain release ATP/adenosine via multiple pathways in physiology. However, ATP/adenosine release and their metabolisms are dysregulated in pathological conditions such as seizure, neurodegenerative disorders, and mental disorders (Illes et al., 2019; Koizumi, 2021; Pietrowski et al., 2021). Here, we discuss ATP/adenosine dynamics in disease with a focus on experimental seizure models, because, in seizure models, activity-dependent release of ATP/adenosine is substantially altered, which is the main focus in this review. Accumulating evidence suggests that purinergic signaling contributes to abnormalities in neuron–glial communication, which underlie the pathogenesis of epilepsy (Rassendren and Audinat, 2016; Beamer et al., 2021). In general, adenosine shows anticonvulsant effect, while ATP shows proconvulsant effect in seizure models (Beamer et al., 2021). The A_1_ receptor is the main target of the anticonvulsant effect of adenosine, while several P2 receptors are targets of the proconvulsant effects of ATP, such as P2X7, P2X4 and P2Y_1_ receptors (Beamer et al., 2021). However, the mechanisms contributing to ATP and adenosine elevations during seizure are not clear. Experiments using enzyme-based biosensors show distinct ATP and adenosine dynamics in experimental seizure and ischemia models (Frenguelli et al., 2007; Lopatář et al., 2011). The changes in ATP are much smaller, and the temporal profiles of the increases differ. For example, in ischemic conditions, ATP elevation starts after the adenosine elevation (Frenguelli et al., 2007).

ATP elevations may occur locally, making measurements using enzyme-based biosensors on the surface of the brain tissue difficult. ATP release may occur locally in health (Hatashita et al., 2023) and disease states (Wu et al., 2022b; Chen et al., 2022), and the rapid degradation of ATP (Dunwiddie et al., 1997) may limit the spread of ATP into the extracellular milieu. Recently, our group found that, in an Alexander disease model, microglia display hyper-ramification and frequent Ca^2+^ signals, both of which are mediated by P2Y_12_ receptor activated by ATP presumably originate from astrocytes. Antagonism of P2Y_12_ receptor worsened the pathological markers, suggesting that microglia play a protective role in the Alexander disease model via P2Y_12_ receptors (Saito et al., 2023) (Figure 1D2). Although we obtained functional evidence for P2Y_12_ receptor activation, we failed to detect local release of ATP with the GRAB_ATP1.0_ sensor. The local release of ATP might be under the detection limit of the GRAB_ATP1.0_ sensor.

Does the comparatively small ATP increase imply that it plays a minor role in seizure pathogenesis? Recent observations suggest that the channel-mediated ATP release pathway may play a key role human epilepsy. Dossi et al. found that pannexin-1 channel activity was increased in brain tissue samples from epileptic patients. Pharmacological interventions targeting pannexin-1 or P2 receptors block interictal discharges in slices. Furthermore, ATP secretion into aCSF is reduced by pannexin-1 blockade (Dossi et al., 2018). These results suggest that ATP increase through pannexin-1 plays an important role in ictal epileptic discharges. Notably, selective depletion of astrocytic pannexin-1 revealed that ATP released through astrocytic pannexin-1 is converted to adenosine, in turn inhibiting neurons, which is an outcome opposite to that produced by global blockade of pannexin-1 (Vasile et al., 2022). Given that pharmacological blockade of pannexin-1 inhibits both neuronal and astrocytic pannexin-1 (Dossi et al., 2018), the contribution of astrocytic pannexin-1 could be minor. A connexin hemichannel-mediated ATP release pathway may also contribute to seizure pathogenesis. A newly developed connexin hemichannel blocker, D4, reduces neuroinflammation, glial change and abnormality of GABAergic synapses in the pilocarpine model of epilepsy (Guo et al., 2022). However, ATP release was not assessed in this study. It would be interesting to investigate whether these mechanisms are specific to seizure or shared with other neurological diseases.

It is currently unclear why so many pathways regulate ATP/adenosine dynamics. However, each mechanism may be recruited by distinct biological and biophysical events in the brain, such as changes in neuronal excitability, osmolality, pH, temperature, and mechanical pressure. In hippocampal slices, studies using radioactive ATP show that high-frequency electrical stimulation induces the release of adenosine that is mainly derived from ATP, compared with low-frequency stimulation, suggesting that high-frequency neuronal activity may preferentially release ATP (Cunha et al., 1996).

As discussed above, ATP release via connexin is linked to mechanosensors. Some of the channel pathways may be associated with a hyperexcitable state. Although the ATP release mechanism is not clear, hyperexcitation of neurons may cause the opening of pannexin-1 under conditions such as low O_2_, elevated extracellular K^+^, mechanical pressure, elevated intracellular Ca^2+^, and high reactive oxygen species levels (Dossi and Rouach, 2021; Weilinger et al., 2023). Changes in CO_2_ and metabolic substrates may also be linked to ATP/adenosine release (Dulla et al., 2005; Huckstepp et al., 2010; Kawamura et al., 2010).

Purine metabolism may also play an important role in adenosine/ATP release. Adenosine content in cells is a key factor in determining how much adenosine is released in response to a stimulus. Adenosine is depleted by repetitive stimuli as well as repeated exposure to hypoxia (Pearson et al., 2001; Klyuch et al., 2011). Adenosine kinase is a key enzyme that metabolizes intracellular adenosine, thereby controlling extracellular adenosine levels. Adenosine kinase is overexpressed in human temporal lobe epilepsy patients (Vezzani et al., 2022), resulting in adenosine deficiency.

Based on the available evidence, one might speculate that ATP release requires drastic changes in the microenvironment. There are three likely mechanisms that mediate activity-dependent ATP/adenosine release. First, short-term or low-frequency pulses may preferentially trigger neuronal adenosine release, which contributes to feed-forward inhibition. Second, long-term pulses or high-frequency pulses may trigger the release of ATP from neurons and astrocytes (and microglia) through multiple pathways. The released ATP is immediately converted to adenosine to contribute to the inhibition of synaptic transmission. Third, in the case of seizure, long-lasting high-frequency pulses might trigger much greater ATP release, thereby surmounting the anti-convulsant effect of adenosine, leading to uncontrolled seizure.

## Conclusion

ATP and adenosine are released from virtually every cell type in the brain, via multiple pathways. ATP and adenosine can exert both excitatory and inhibitory effects on neuronal activity. Many receptors, channels, transporters, and enzymes that regulate ATP/adenosine are shared across multiple cell types. The neuronal response to the ATP/adenosine elevation depends on the expression and localization of numerous molecules involved in purinergic signaling. Therefore, direct measurement of the spatiotemporal dynamics of ATP/adenosine at the subcellular level is essential for uncovering how neurons and glia communicate with each other via purinergic signaling. Furthermore, simultaneous monitoring of other modalities (such as Ca^2+^, H^+^, voltage, and cAMP) with direct measurement of ATP/adenosine is useful to advance our understanding of the function of purinergic signaling in neuron-glia communication. Tools that allow for the analysis of ATP/adenosine release with high spatiotemporal resolution are needed to advance our understanding of how purinergic signaling regulates neuron–glial networks in health and disease.
